# Vaccine-induced donor-specific HLA antibodies: a case report highlighting sensitization risks in renal transplant waitlisted patients

**DOI:** 10.3389/fimmu.2025.1567377

**Published:** 2025-03-14

**Authors:** Pramath Kakodkar, Nooshin Shekari, Rahul Mainra, Destinie Webster, Twyla Pearce, Fang Wu, Ahmed Mostafa

**Affiliations:** ^1^ Department of Pathology and Laboratory Medicine, University of Saskatchewan, Saskatoon, SK, Canada; ^2^ Department of Anatomy, Physiology and Pharmacology, University of Saskatchewan, Saskatoon, SK, Canada; ^3^ Division of Nephrology, Department of Medicine, University of Saskatchewan, Saskatoon, SK, Canada; ^4^ Histocompatibility and Immunogenetics Laboratory, St. Paul’s Hospital, Saskatoon, SK, Canada

**Keywords:** donor-specific HLA antibodies, pneumococcal vaccine, zoster vaccine, renal transplant, vaccination in kidney transplant, vaccines induced HLA antibodies

## Abstract

**Background:**

In renal transplant waitlisted patients, vaccinations remain the standard of care for infection prevention. The vaccine and its adjuvant sensitizer can be potential sources for the induction of donor-specific antibodies (DSA) against human leukocyte antigens (HLA). These novel HLA antibodies can result in a positive flow cell crossmatch (FCXM), which can make a previously compatible live donor incompatible.

**Case report:**

We present an adult renal transplant waitlisted patient who has had multiple negative T-cell and B-cell FCXM with no detection of DSA at baseline. The patient then received a single dose of pneumococcal conjugate (PCV13) and a second dose of recombinant zoster vaccine (RZV). After these vaccinations, the patient’s FCXM was positive for both T-cells and B-cells and the HLA class I antibodies (A1, 23, 24, 80; B44, 45, 76) showed a calculated panel reactive antibody (cPRA) of 51%. A1 and B44 DSA were detected which predicted incompatibility with the patient’s planned live donor renal transplant. The patient had to enter the kidney-paired donation program instead and receive their transplantation after 16 months.

**Conclusion:**

RZV or PCV13 vaccines or their adjuvant components can potentially cause allosensitization in renal transplant waitlisted patients. The detection of DSA can result in reduced access to compatible transplants. With advances in HLA immunogenetics, better tools can monitor HLA-specific memory B-cells to provide crucial insights into the primary mechanism of action of HLA DSA antibody formation and suggest interventions to mitigate this memory B-cell activation.

## 1 Introduction

Infection prevention remains paramount for the optimization of renal transplant patients on the waitlist. Many of these patients with chronic kidney disease (CKD) have progressed to end-stage renal disease (ESRD) with suboptimal innate and adaptive immune responses (1, 2). The Kidney Disease: Improving Global Outcomes (KDIGO) clinical best practice guideline for renal transplant candidates recommends a comprehensive vaccination series before kidney transplantation (3). This vaccination series aims to protect these patients from pre-transplant and immediately post-transplant. KDIGO and the Infectious Disease Society of America (IDSA) caution against the usage of live attenuated vaccinations (mumps, measles, rubella, varicella, and intranasal influenza) within 4 weeks from transplantation due to the theoretical possibility of the proliferation of the attenuated organism (3, 4). Therefore, the pretransplant vaccination series is instituted widely to protect these patients against vaccine-preventable illnesses such as COVID-19, pneumococcal infections, influenza, varicella zoster, hepatitis A, and hepatitis B (5).

Although the vaccination series in renal transplant waitlisted patients remains the standard of care, there is a need to investigate if any of these vaccines can induce donor-specific Antibodies (DSA) against human leukocyte antigens (HLA). These novel HLA antibodies (HLA abs) in renal transplant waitlisted patients can result in a positive flow cell cross-match (FCXM), which would be suggestive of an increased risk of renal transplant rejection and also reduce access to compatible transplants (6).

The literature suggests that COVID-19, seasonal influenza, and pneumococcal vaccines can produce HLA abs in renal transplant waitlisted patients (6–9). Despite the generation of these DSA, there were few documented cases of solid organ rejection due to COVID-19, seasonal influenza, and pneumococcal vaccines. Nevertheless, the identification of DSA during pretransplant assessment could lead to delays in access to compatible transplantation for the waitlisted transplant candidates and potentially increase the risk of renal transplant rejection. In this article, we presented a case report on a renal transplant waitlisted patient with minimal history of sensitization, developing DSA following pneumococcal conjugate (PCV13) and recombinant zoster vaccination (RZV) vaccination.

## 2 Case presentation

Patient was initially diagnosed with obstructive nephropathy and hypertension. The patient then progressed to end-stage renal disease requiring transplantation and was initially selected to receive a living donor kidney transplant from their sibling. The patient’s sensitization history was notable for a blood transfusion in 2016. The patient and donor (sibling) were confirmed to share a matched ABO blood group O. Subsequently, low-resolution HLA typing was performed via reverse sequence-specific oligonucleotide (SSO) for HLA class I (HLA-A; B; C) and HLA Class II (HLA-DRB1; DQA1, DQB1; DPA1, DPB1) (**Supplementary Table 1**).

**Figure 1:**
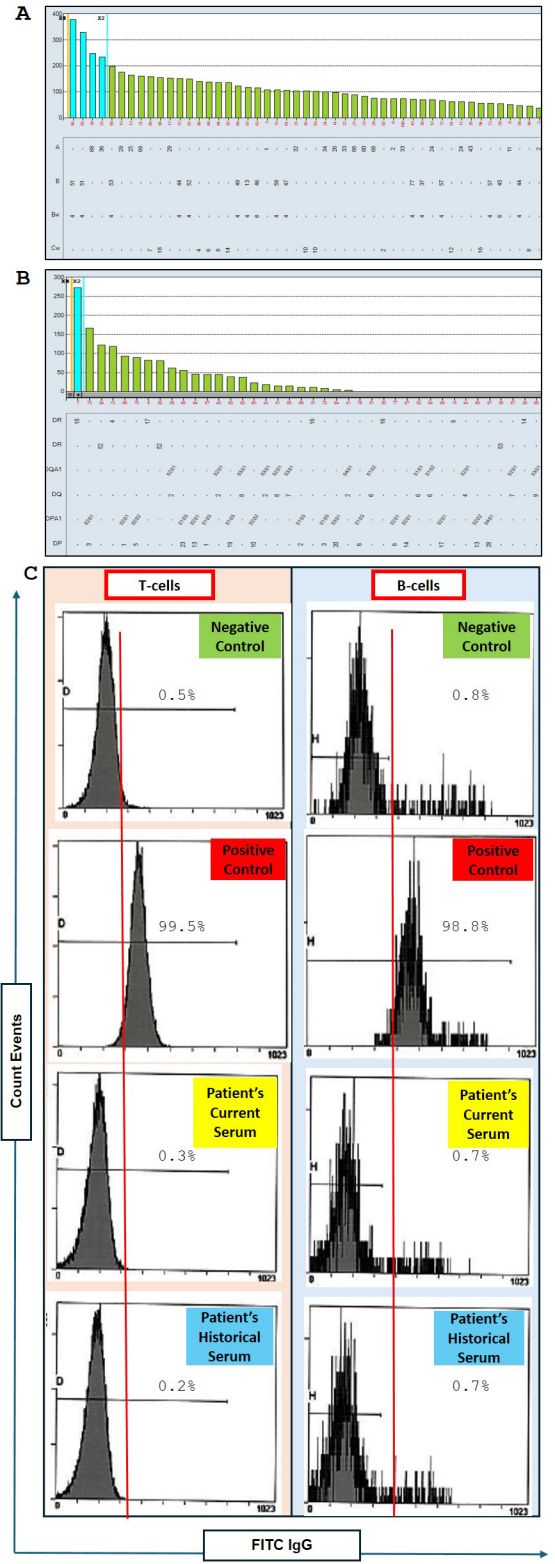
Assessment of donor-specific anti-HLA antibodies and flow cytometry crossmatch (FCXM) results. **(A)** Luminex LABScreen single antigen bead (SAB) analysis of HLA antibodies targeting HLA Class (I) The patient’s baseline sample showed no reactivity against Class I HLA antigens. The cutoff is <1000 MFI. **(B)** Luminex LABScreen SAB analysis of HLA antibodies targeting HLA Class II. No detectable antibodies were found prior to vaccination. The cutoff is <1000 MFI. **(C)** Flow cytometry crossmatch (FCXM) results comparing the patient’s current serum to a historical serum sample collected one year prior. Both sera are evaluated against negative and positive controls for T-cell and B-cell assays using fluorescein isothiocyanate-conjugated anti-IgG (FITC IgG) to detect anti-HLA antibodies specific to donor T cells and B cells. No reactivity was observed prior to vaccination.

**Supplementary Table 1** shows that 4 out of 6 donor HLA antigens were mismatched for A1, 2; B44; and DR4. To evaluate the HLA antibody in the patient’s serum, an HLA antibody screen was performed using a single antigen bead (SAB) assay. **Figures 1A, B** shows no reactivity against HLA class I and II antigens with a 0% calculated panel reactive antibody (cPRA). Our institutional threshold of mean fluorescence intensity (MFI) for positivity was set at ≥1000.

We conducted a flow cell crossmatch (FCXM) during the initial assessment phase. The crossmatch was performed using the patient’s current serum (the most recent sera tested for SAB) and historical serum from a year prior. As illustrated in **Figure 1C**, both these sera were negative for T-cell and B-cell crossmatch. The recipient and potential donor were deemed compatible for standard risk to proceed with transplantation based on the results from HLA typing, HLA antibody screen, and FCXM.

The corresponding quarterly serum samples remained negative for both HLA class I and II antibodies. However, the transplant surgery was postponed due to recurrent urinary tract infections (UTIs) caused by reflux. Subsequently, the patient underwent a transurethral resection of the prostate (TURP) procedure. Following the TURP procedure, the patient has not experienced recurrent infections for over a year. Thirteen months after the TURP and UTI episodes, the sera collected from the patient continued to be negative for T-cell and B-cell crossmatch.

**Figure 2:**
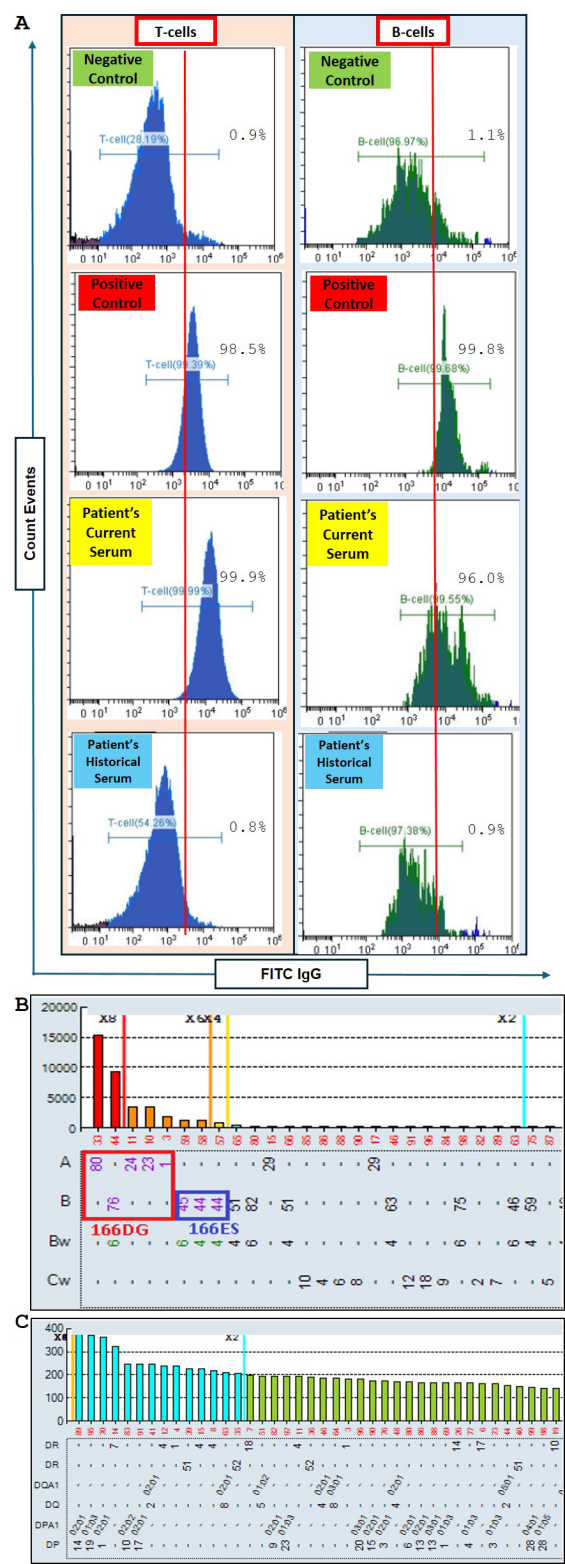
Flow cytometry crossmatch (FCXM) and Luminex LABScreen single antigen bead (SAB) screening for anti-HLA antibodies. **(A)** FCXM results comparing the patient’s current serum after pneumococcal conjugate (PCV13) and recombinant zoster vaccination (RZV) to non-vaccinated historical serum. The sera are evaluated against negative and positive controls for T-cell and B-cell assays. Fluorescein isothiocyanate-conjugated anti-IgG (FITC IgG) is used to detect anti-HLA antibodies targeting donor T cells and B cells. Increased reactivity is observed post-vaccination. **(B)** Luminex LABScreen single antigen bead (SAB) analysis of anti-HLA antibodies targeting HLA Class I, with epitope analysis highlighting the 166DG and 166ES antigens forming a cross-reactive antigen group. The post-vaccination sample showed increased MFI values (cutoff <1000 MFI), particularly for A1 and B44, suggesting new DSA emergence. **(C)** Luminex LABScreen SAB analysis of anti-HLA antibodies targeting HLA Class II. No new Class II antibodies were detected post-vaccination. The cutoff is <1000 MFI.

As per our agreement with the transplant center, the flow cytometry crossmatch (FCXM) is generally performed annually until the transplant procedure is completed. Furthermore, a final crossmatch is carried out approximately two weeks before the scheduled surgery. When the transplant surgery was rescheduled, FCXM testing was repeated to assess any changes from the previous baseline FCXM. Surprisingly, the repeat FCXM showed positive reactivity for both T and B cells (**Figure 2A**), indicating the presence of DSA either against HLA class I or both HLA class I and II.

**Figure 3:**
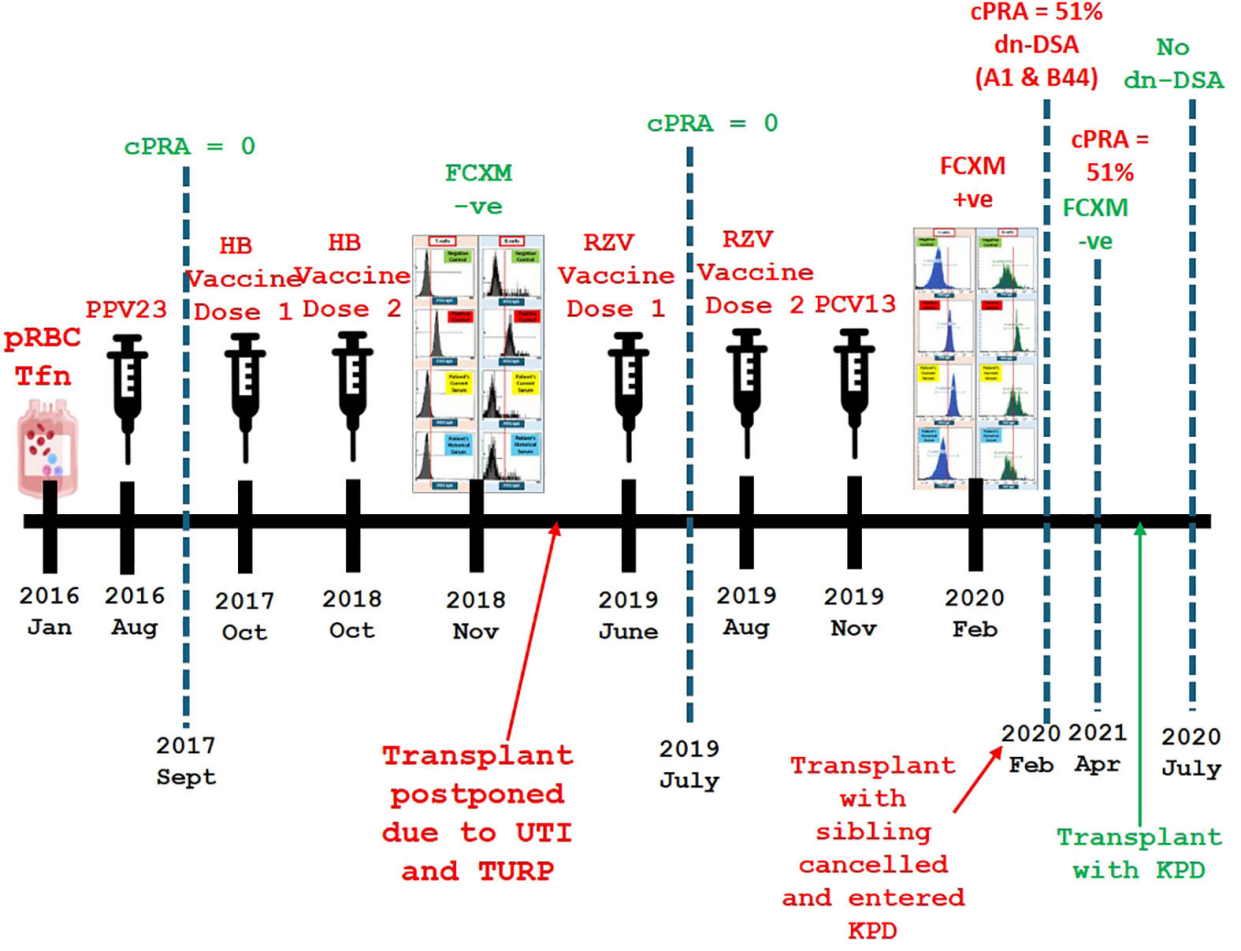
Timeline of sensitization events and pre-transplant immunological assessments. This figure provides a chronological summary of sensitization events, including prior blood transfusion, urinary tract infections (UTIs), transurethral resection of the prostate (TURP), and vaccinations. It also outlines the timing of anti-HLA surveillance and corresponding changes in calculated panel reactive antibody (cPRA) levels, flow cytometry crossmatch (FCXM) results, and *de novo* donor-specific antibody (dn-DSA) detection. Calculated Panel Reactive Antibody (cPRA) is a measure used to estimate the likelihood of a transplant candidate having pre-existing antibodies against a potential donor’s HLA antigens. Kidney Paired Donation (KPD) is a transplant program that matches incompatible donor-recipient pairs with other pairs to enable a compatible kidney exchange. packed red blood cells (pRBC), transfusion (Tfn), pneumococcal polysaccharide 23 (PPV23), hepatitis B (HB), recombinant zoster vaccine (RZV), pneumococcal conjugate vaccine 13 (PCV13), urinary tract infection (UTI), transurethral retrograde prostatectomy (TURP), panel reactive antibody score (cPRA), flow cytometry crossmatch (FCXM), *de novo* donor-specific antibody (dsDSA), kidney paired donor program (KPD), negative (-ve).

To confirm this finding, SAB on the repeat FCXM positive sample was performed. SAB testing for HLA class II showed no evidence of *de novo* HLA class II antibodies (**Figure 2C**). However, the HLA class I antibody screening test detected the presence of antibodies for A1, 23, 24, 80; B44, 45, 76 (cPRA of 51%) with DSA targeting A1 and B44 (**Figure 2B**). These findings suggested that a novel sensitizing event had occurred since the last cPRA of 0% which was performed 216 days prior. Upon reviewing the patient’s clinical history, it was found that the patient had received a single dose of the pneumococcal conjugate vaccine (PCV13) 76 days prior and the second dose of the recombinant zoster vaccine (RZV) 162 days prior. Additionally, the patient had been vaccinated against hepatitis B, seasonal influenza, pneumococcal polysaccharide 23 (PPCV23), and the first dose of RZV before the last cPRA of 0%. A summary of the possible sensitizing events and the timeline of anti-HLA surveillance is shown in **Figure 3**.

Although the mean fluorescence intensity (MFI) for DSA was 1800 for A1 and 1300 for B44, the T cell FCXM reaction was significantly stronger than the positive control sample. This strong T-cell crossmatch suggested an epitope spread, prompting an epitope analysis to identify any shared epitopes that might explain the antibody cross-reactivity pattern. Epitope analysis highlighted 166DG and 166ES as the shared epitopes responsible for the entire specificity (**Figure 2B**). The breadth of HLA antibodies increased primarily within the same cross-reactive antigen group (CREG), indicating an expansion of existing specificities without the development of new ones. The A1 dn-DSA had an MFI of 1800 and shared the same epitope with A80, with an MFI of 15,000. This could explain the strong positivity observed in the T cell and B cell FCXM.

Due to the presence of those DSA, the patient’s live donor renal transplant with their sibling became classified as high risk for transplant rejection. Consequently, the patient was now placed in the kidney-paired donation (KPD) program to find a new compatible donor. At this time, the repeat antibody screen before the KPD transplant surgery remained unchanged (cPRA 51%), which indicated persistent sensitization. The FCXM with the new KPD patient was B-cell and T-cell negative, which confirmed compatibility and a low risk of rejection. The patient underwent transplant surgery via the KPD program after a 16-month delay since the previous planned transplant. One month after the kidney transplantation, the antibody screen remained unchanged with no evidence of DSA (Data not shown).

At the 40-month follow-up post-transplant, the patient did not develop DSA or any sort of rejection. The patient’s blood creatinine levels were stable, ranging between 110-125 µmol/L, within the normal range of 60-130 µmol/L.

## 3 Discussion

Our case report highlights the development of HLA DSA in a renal transplant waitlisted patient after receiving a single dose of PCV13 and two doses of RZV. Our patient’s planned live donor renal transplant with their sibling was halted, and they had to enter the KPD program. This entire process delayed their transplantation by 16 months. The recent COVID-19 pandemic has highlighted the importance of vaccination series in optimizing infection prevention within renal transplant waitlisted patients (10). Contrastingly, the literature also shows that COVID-19, seasonal influenza, and pneumococcal vaccines can produce anti-HLA abs in renal transplant waitlisted patients (6–9, 11, 12). However, the presence of DSA in our patient cannot be definitively attributed to vaccination alone. The patient had a prior blood transfusion in 2016, a known risk factor for alloimmune sensitization, which may have induced latent alloimmune memory. Additionally, the patient’s history of recurrent UTIs and subsequent TURP procedure represent immune-stimulating events that could have contributed to an immune response leading to DSA emergence. It is possible that these pre-existing sensitization factors, combined with vaccination, collectively influenced the development of *de novo* DSA. Despite these potential confounders, the timing of vaccination and subsequent DSA emergence strongly suggest a causal link, making vaccination the most plausible sensitizing event. The detection of DSA occurred shortly after the administration of PCV13 and RZV, indicating a temporal association. This aligns with existing literature that reports the generation of HLA antibodies following vaccination in renal transplant waitlisted patients. Therefore, while it is important to acknowledge the role of other sensitizing events, vaccination remains the most likely cause of DSA formation in this case.

**Figure 4:**
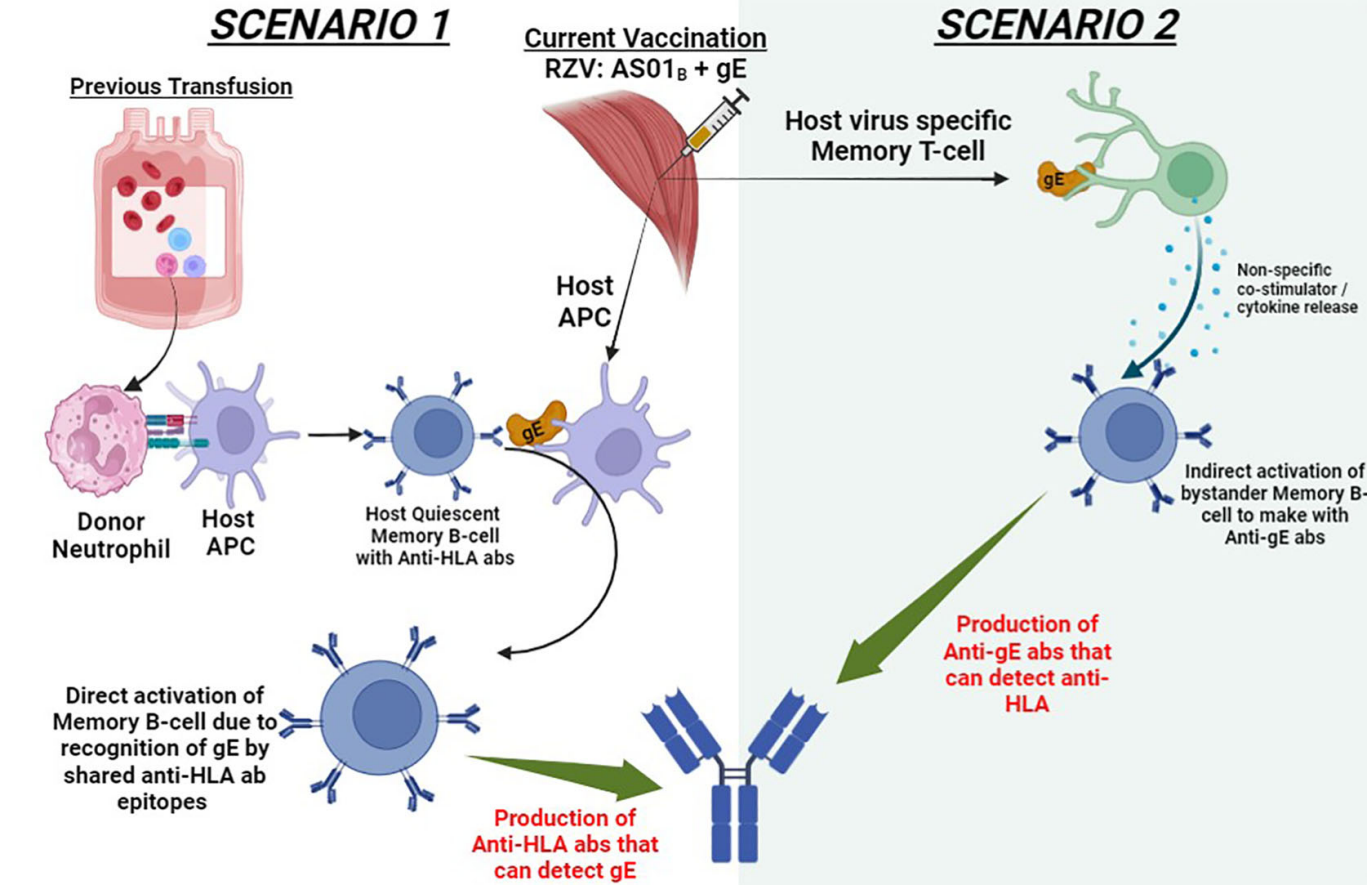
Proposed mechanisms for dn-DSA anti-HLA antibody formation following intradeltoid recombinant zoster vaccine (RZV) administration. This schematic illustrates two potential pathways for dn-DSA development: (1) Activation of quiescent memory B-cells due to prior alloimmune sensitization events such as blood transfusion, facilitated by the immunogenic effects of vaccine adjuvants. (2) A bystander activation mechanism in which vaccine-induced immune responses generate cross-reactive antibodies that recognize HLA epitopes. Adjuvants such as AS01_B_ may play a role in enhancing immune activation through Toll-like receptor signaling and antigen-presenting cell recruitment. These mechanisms highlight the potential immunological pathways linking vaccination to alloimmune responses in transplant candidates. This figure illustrates a theoretical model based on current hypotheses rather than established evidence. adjuvant suspension (AS01_B_), VZV glycoprotein E (gE), antigen-presenting cells (APC).

The schematic diagram in **Figure 4** outlines the two possible scenarios that could explain the production of HLA DSA in our patient after RZV vaccination. In scenario 1, the patient’s previous blood transfusion could have created the now quiescent memory B-cell against any of the following HLA (A1, 23, 24, 80; B44, 45, 76). After vaccination with RZV, the adjuvant AS01_B_ composed of Toll-like receptor 4 (TLR4) agonist and monophosphoryl lipid A (MLP) causes a robust host antigen-presenting cell (APC) recruitment (13). The potentially shared epitopes, such as VZV glycoprotein E (gE) can be presented on the APC on the membrane-bound anti-HLA abs of the quiescent memory B-cell. This model of anamnestic B-cell response due to heterologous immunity has been documented in the literature (14, 15). Contrastingly, in scenario 2, the intramuscular RZV stimulates the host virus-specific memory T cell to detect the gE. This detection of gE results in the release of multiple co-stimulators or cytokines, which activate bystander memory B-cells to produce anti-gE abs. These anti-gE abs produced by memory B-cell clonotypes can have molecular mimicry wherein they may potentially share the predominant epitopes 166DG and 166ES in our donor, yielding an anti-HLA cross reactivity. This relationship between viral immune response and anamnestic B-cell responses has been described in the literature (16–18). While we propose that adjuvant-mediated immune activation, particularly via the AS01_B_ adjuvant in RZV, contributed to DSA formation, we acknowledge the lack of experimental validation to confirm this mechanism. No B-cell ELISPOT assays, cytokine profiling, or *in vitro* T-cell activation studies were conducted to demonstrate this process. Future studies should incorporate these methodologies to better elucidate the immunological pathways involved in vaccine-associated sensitization. Additionally, Potential confounders such as prior blood transfusions, urinary tract infections, and the TURP procedure may have contributed to sensitization. Blood transfusions introduce foreign HLA antigens, potentially triggering HLA antibody formation and inducing latent alloimmune memory, which subsequent immune challenges like vaccination could reactivate. Urinary tract infections and the TURP procedure may have further influenced sensitization by activating memory B cells, triggering antibody production, and causing immune stimulation through inflammation. Considering these factors provides a more comprehensive interpretation. While vaccination remains the most likely sensitizing event based on DSA timing, other contributors should not be overlooked. Similar scenarios can be reconstructed with the patient being vaccinated, resulting in antibody formation against capsular polysaccharide of 13 pneumococcal serotypes. It is interesting to note that the patient first received a single dose of PPCV23 three years prior with no corresponding increase in DSA anti-HLA abs. It was only when they received the PCV13 that they developed DSA anti-HLA abs. Interestingly, the comprehensive safety profile of PPCV23 and pneumococcal conjugate 7 (Pneu-C-7) vaccinations in these kidney transplant patients has been well-established (19). Contrastingly, only a small study in kidney transplant recipients was conducted to evaluate the formation of dn-DSA or anti-HLA abs after PCV13 vaccination. In this study, only a few patients (33.3%, n=5 out of 15) developed HLA abs, but none produced DSA (20). It is important to note that these findings cannot be generalized as only a minor cohort of these PCV13 vaccinated patients (33.3%, n=15 out of 45) underwent HLA abs testing (20).

In our case report, PCV13 vaccination is a possible trigger for DSA due to its heightened immunogenicity, driven by the conjugated diphtheria CRM197 protein carrier. The conjugate vaccine, such as PCV13, needs to combine weaker immunogenic antigens, such as Streptococcus pneumonia capsular polysaccharides, with stronger immunogenic adjuvants, such as adsorbed CRM197 protein carriers on aluminum phosphate (21). A modified double-blind randomized trial showed that PCV13 has potent immunogenicity compared to PPCV23, as evidenced by the anti-pneumococcal opsonophagocytic activity (21). This increased immunogenicity of PCV13 would have led to a higher propensity to develop dn-DSA anti-HLA abs.

In this case report, the patient received both PCV13 and the 2^nd^ dose of RZV just before the antibody screening test, which resulted in a cPRA of 51%. As such, we are unable to pinpoint which vaccine or vaccine component was the primary inducer of these HLA DSA. Alternatively, if both vaccines or vaccine components were inducers, then we could not quantify the weightage of induction of HLA DSA by each vaccine component. Notably, scenario 2 (**Figure 4**) *in vitro* studies have shown a heightened fold increase in the MFI values similar to our patient (22). As such, scenario 2, with RZV as the potential causative agent, is favored as the underlying mechanism for dn-DSA anti-HLA formation.

The risk to transplant success continues to be substantiated by further evidence, it raises the question of whether the risk of allosensitization secondary to a particular vaccination is a recognized risk to transplant recipients. With advances in HLA immunogenetics, better tools to monitor HLA-specific memory B-cells will provide crucial insights into the primary mechanism of action of HLA DSA formation and suggest interventions to mitigate this memory B-cell activation.

The case presented involves an adult patinet, but it is important to consider how vaccine-induced donor-specific HLA antibodies might affect children, who typically receive more vaccines. In children, the immune system is still developing, and they receive a higher number of vaccines compared to adults (23). This increased exposure could theoretically lead to a higher risk of developing DSA. However, the literature on vaccine-induced HLA antibodies in children is limited. One relevant review by Rees and Kim (2014) discusses HLA sensitization, which can occur after transfusion of blood products, transplantation, and spontaneously through cross-sensitization from infections and pro-inflammatory events (24). The review highlights that children are particularly vulnerable to HLA sensitization due to their likelihood of needing multiple transplants over their lifetime. It also mentions concerns about the potential for HLA allo-sensitization following vaccinations. While some studies have detected HLA antibodies post-vaccination, the clinical significance of these findings is not well established. Most detected HLA antibodies declined or disappeared on follow-up testing, and other studies have not shown an adverse effect of vaccinations on HLA sensitization. Established vaccinations remain recommended for children with chronic kidney disease (CKD). In a study of a pediatric population of 23 children awaiting transplants who did not receive blood products, 26% developed HLA antibodies over 19 months (25). This suggests that sensitization could occur due to various factors, including vaccinations and infection.

Therefore, solid organ transplant waitlisted patients and transplant teams must be made aware of the potential risk of developing DSA following certain vaccinations. Furthermore, this risk must be weighed against the benefits of the vaccine-induced antibody-mediated immunization in preventing infections during their post-transplant phase. Therefore, shared decision-making between these patient groups and the transplant teams is vital to understand the clinical weightage of these factors and to acknowledge the risks and benefits of vaccination in the context of their candidacy for a successful transplantation.

In our case report, previous hepatitis B and seasonal influenza vaccinations were ruled out as the causative agent in the induction of these HLA DSA via antibody screening or FCXM in the patient’s serum sample before PCV13 and the 2nd dose of RZV vaccination. Interestingly, the majority of transplant recipients are successfully vaccinated without the development of DSA. The background sensitization history or HLA profile could be the underlying factors that increase the risk of vaccine-induced alloimmunization in a subset of individuals. The key takeaway is that we advised our transplant center to consider vaccination as a potential sensitizing event. We strongly recommend performing HLA antibody testing three weeks post-vaccination to detect any development of HLA antibodies.

## 4 Conclusion

This case underscores the potential for vaccinations to be associated with, but not necessarily causative of, DSA emergence in renal transplant waitlisted patients. While prior hepatitis B and seasonal influenza vaccinations were ruled out as direct contributors to sensitization, the concurrent administration of Pneu-C-13 and the second dose of RZV before the DSA detection raises important considerations. It is crucial to recognize that other immune-stimulating events, including a history of blood transfusion, UTIs, and TURP, may have played a role in sensitization (26).

Given the complexity of alloimmune responses, future studies should explore the interplay between vaccinations, pre-existing immune memory, and donor-specific antibody formation. Advances in HLA immunogenetics and the ability to monitor memory B-cell responses could provide valuable insights into the mechanisms driving DSA development and inform strategies to mitigate unintended sensitization in transplant candidates. Recognizing these limitations provides a comprehensive understanding of the case, supporting the conclusion that vaccination is the most plausible cause of DSA emergence.

## Data Availability

The original contributions presented in the study are included in the article/**Supplementary Material**. Further inquiries can be directed to the corresponding author.

## Supplementary material

Supplementary Table 1 Summary of HLA typing results for Patient and Sibling (donor).
