# Supplementary material for: Vaccine-induced donor-specific HLA antibodies: a case report highlighting sensitization risks in renal transplant waitlisted patients
Source: Front Immunol. 2025 Mar 14;16:1567377. doi: 10.3389/fimmu.2025.1567377 (PMC11949935; doi:10.3389/fimmu.2025.1567377)
Supplement: Supplementary Table 1 — Summary of HLA typing results for Patient and Sibling (donor). [file Table1.docx]

1. **Supplementary Table**

**Supplementary Table 1:** Summary of HLA typing results for Patient and Sibling (donor)

|  |  | A* | B* | C* | DRB1* | DRB3/4/5 | DQA1* | DQB1* | DPA1* | DPB1* |
| --- | --- | --- | --- | --- | --- | --- | --- | --- | --- | --- |
| Patient | a | 03:01 | 07:02 | 02:02 | 11:01 | 3*02:02 | 01:02 | 03:01 | 01:03 | 04:01 |
|  | c | 03:01 | 40:02 | 07:02 | 15:01 | 5*01:01 | 05:05 | 06:02 | 01:03 | 04:01 |
| Donor  (Sibling) | b | 01:01 | 44:02 | 05:01 | 04:01 | 4*01:01 | 03:01 | 03:01 | 01:03 | 04:01 |
|  | d | 02:01 | 44:02 | 05:01 | 04:01 | 4*01:01 | 03:01 | 03:01 | 01:03 | 04:01 |
